# Supplementary figures and images for: Trends and determinants of prelacteal feeding practice in rural Bangladesh from 2004 to 2019: A multivariate decomposition analysis
Source: PLoS One. 2026 Mar 11;21(3):e0328943. doi: 10.1371/journal.pone.0328943 (PMC12978494; doi:10.1371/journal.pone.0328943)

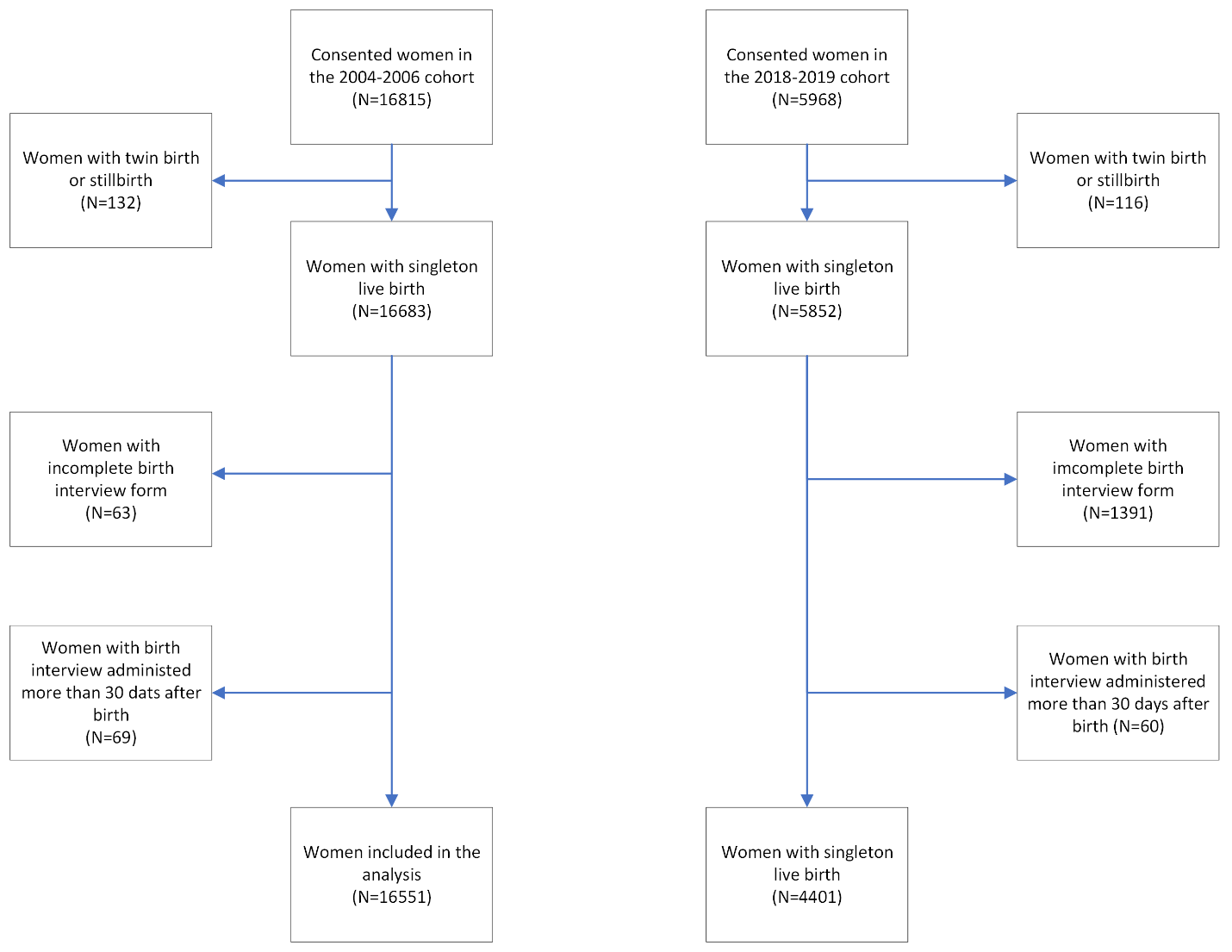

Supplement: S1 Fig — (TIF) [file pone.0328943.s001.tif]
