# Supplementary material for: Trends and determinants of prelacteal feeding practice in rural Bangladesh from 2004 to 2019: A multivariate decomposition analysis
Source: PLoS One. 2026 Mar 11;21(3):e0328943. doi: 10.1371/journal.pone.0328943 (PMC12978494; doi:10.1371/journal.pone.0328943)
Supplement: S1 Table — (PDF) [file pone.0328943.s002.pdf]

**S1 Table. Disaggregated results for Oaxaca-Blinder decomposition analysis of change in prelacteal feeding prevalence in Bangladesh, 2004-2019**

| Characteristics | Share of total change, % (Explained)[1] | Share of total change, % (Unexplained)[2] |
|---|---|---|
| Maternal age | | |
| ⩽19 | 0 | 0 |
| 20-34 | 0.71* | -6.52* |
| ⩾35 | 0.01 | -0.57 |
| Maternal literate | | |
| Yes | 0 | 0 |
| No | 0.29 | -2.35 |
| Parity | | |
| Primigravid | 0 | 0 |
| Multigravid | 1.78* | -2.29 |
| Participation to micro-credit program | | |
| Yes | 0 | 0 |
| No | 0.21 | -0.30 |
| Infant sex | | |
| Male | 0 | 0 |
| Female | -0.03 | 1.68 |
| Birth location | | |
| Home† | 0 | 0 |
| Health Facility‡ | 9.06* | -13.54* |
| Type of delivery | | |
| Vaginal | 0 | 0 |
| Cesarean | 0.80 | -1.27 |
| Birth weight | 1.84* | -18.39* |
| Constant | | 128.91* |
| Overall | 14.65 | 85.35 |

*P value <0.05.

†Includes home and enroute/other, number of births happened enroute/other is much smaller than number of births happened at home, See Table 1.

‡Includes family welfare visitor's houses; or health or welfare center; or Hospital/clinic/medical college.

[1] The explained component refers to changes in PLF prevalence accounted for by changes in the means of the explanatory variables multiplied by their corresponding regression coefficients from Table 3.

[2] The unexplained component consists of two parts: variations in regression coefficients between baseline and endline; and the interaction between changes in coefficients and changes in explanatory variables.
