# Supplementary material for: Trends and determinants of prelacteal feeding practice in rural Bangladesh from 2004 to 2019: A multivariate decomposition analysis
Source: PLoS One. 2026 Mar 11;21(3):e0328943. doi: 10.1371/journal.pone.0328943 (PMC12978494; doi:10.1371/journal.pone.0328943)
Supplement: S2 Table — (PDF) [file pone.0328943.s003.pdf]

S2 Table. Disaggregated results for Oaxaca-Blinder decomposition analysis of change in prelacteal feeding prevalence in Bangladesh among primigravida women, 2004-2019

Characteristics	Share of total change, % (Explained)[1]	Share of total change, % (Unexplained)[2]
Maternal age		
⩽19	0	0
20-34	0.21	-3.54
⩾35	0.08	-0.22
Maternal literate		
Yes	0	0
No	0.84	-1.12
Participation to micro-credit program		
Yes	0	0
No	-0.13	6.67*
Infant sex		
Male	0	0
Female	-0.02	4.93
Birth location		
Home†	0	0
Health Facility‡	9.97*	-14.58*
Type of delivery		
Vaginal	0	0
Cesarean	2.85	-3.20
Birth weight	2.37*	-26.95
Constant		121.84*
Overall	16.16	83.84

*P value <0.05.

†Includes home and enroute/other, number of births happened enroute/other is much smaller than number of births happened at home, See Table 1.

‡Includes family welfare visitor's houses; or health or welfare center; or Hospital/clinic/medical college.

[1] The explained component refers to changes in PLF prevalence accounted for by changes in the means of the explanatory variables multiplied by their corresponding regression coefficients from Table 3.

[2] The unexplained component consists of two parts: variations in regression coefficients between baseline and endline; and the interaction between changes in coefficients and changes in explanatory variables.
